# IL-1β Inflammatory Cytokine-Induced *TP63* Isoform ∆NP63α Signaling Cascade Contributes to Cisplatin Resistance in Human Breast Cancer Cells

**DOI:** 10.3390/ijms20020270

**Published:** 2019-01-11

**Authors:** Mónica G. Mendoza-Rodríguez, Jorge T. Ayala-Sumuano, Lázaro García-Morales, Horacio Zamudio-Meza, Eloy A. Pérez-Yepez, Isaura Meza

**Affiliations:** 1 Departamento de Biomedicina Molecular, Centro de Investigación y de Estudios Avanzados del Instituto, Politécnico Nacional, Avenida Instituto Politécnico Nacional 2508, Ciudad de México 07360, Mexico; mgmendoza@cinvestav.mx (M.G.M.-R.); lazaro.garcia@cinvestav.mx (L.G.-M.); drhzamudio@gmail.com (H.Z.-M.); eperezy2306@gmail.com (E.A.P.-Y.); 2 IDIX SA de CV, Sonterra 3035, Querétaro 76235, Mexico; tonatiuh@cinvestav.mx

**Keywords:** IL-1β, *TP63* isoform ΔNP63α, short hairpin RNA (shRNA)-mediated knockdown, drug resistance acquisition, breast cancer

## Abstract

The mechanisms behind the induction of malignancy and chemoresistance in breast cancer cells are still not completely understood. Inflammation is associated with the induction of malignancy in different types of cancer and is highlighted as an important factor for chemoresistance. In previous work, we demonstrated that the inflammatory cytokine interleukin 1β (IL-1β)-induced upregulation of genes was associated with chemoresistance in breast cancer cells. Here, we evaluated the participation and the expression profile of *TP63* in the induction of resistance to cisplatin. By loss-of-function assays, we identified that IL-1β particularly upregulates the expression of the tumor protein 63 (TP63) isoform ΔNP63α, through the activation of the IL-1β/IL-1RI/β-catenin signaling pathway. Upregulation of ΔNP63α leads to an increase in the expression of the cell survival factors epidermal growth factor receptor (EGFR) and phosphatase 1D (Wip1), and a decrease in the DNA damage sensor, ataxia-telangiectasia mutated (ATM). The participation of these processes in the increase of resistance to cisplatin was confirmed by silencing *TP63* expression or by inhibition of the phosphoinositide 3-kinase (PI3K)/protein kinase B (AKT) activity in the IL-1β/IL-1RI/β-catenin signaling pathway. These data reinforced the importance of an inflammatory environment in the induction of drug resistance in cancer cells and uncovered a molecular mechanism where the IL-1β signaling pathway potentiates the acquisition of cisplatin resistance in breast cancer cells.

## 1. Introduction

Interleukin 1β (IL-1β) is an inflammatory cytokine that, either alone or in combination with other factors, favors the maintenance of an inflammatory tumor microenvironment [1]. IL-1β-induced inflammation is considered a risk factor related to the acquisition of an aggressive phenotype in cancer cells [2]. We recently reported, using MCF-7 non-invasive breast cancer cells, selected to homogenously respond to IL-1β (6D cells), that this cytokine induces a signaling pathway that contributes to the activation of β-catenin, overexpression of several genes involved in the epithelial–mesenchymal transition (EMT), and chemoresistance [3,4,5,6]. RNA-sequence (RNA-seq) analyses of IL-1β-stimulated breast cancer cells indicated overexpression of the *TP63* gene, a member of the *P53* suppressor gene family [5]. The *TP63* gene is associated with breast cancer resistance to *cis*-diammine-dichloroplatinum II (cisplatin), a particular drug used for the treatment of solid tumors and aggressiveness [7,8].

The protein codified by *TP63*, called tumor protein 63 (TP63), has at least ten isoforms that differ in the *N*-terminal and *C*-terminal regions as a result of alternative promoter usage or alternative splicing [9]. These proteins are divided into two major groups: the TAp63 and the ΔNp63 isoforms. The TAp63 isoforms have similar activity to that shown by the protein coded by the suppressor gene *P53* that positively regulates cell-cycle arrest and apoptosis [10]. In contrast, the ΔNp63 isoforms promote regulation of pro-survival genes and inhibit expression of cell-cycle arrest genes [11]. ΔNP63α, the most abundant of these isoforms, is highly expressed in various human epithelial cancers [12,13]. Increased expression of ΔNP63α in ovarian and nasopharyngeal carcinomas was reported to increase their cell proliferation, enhance tumor growth, and block p53-dependent apoptosis [12,13]. Nuclear translocation of β-catenin and its binding to the *TP63* promoter was proposed as one of the underlying mechanisms that contributes to ΔNP63α upregulation and tumorigenesis [14]. However, the factors that trigger ΔNP63α expression are still not well characterized.

In the present study, we demonstrate that IL-1β-dependent activation of the IL-1β/IL-1RI/β-catenin signaling pathway [4] induces the acquisition of resistance to cisplatin through upregulation of ΔNp63α, which in turn increases the epidermal growth factor receptor (EGFR) expression, survival signals, and suppression of pro-apoptotic proteins. The high levels of ΔNp63α also altered the phosphatase 1D (Wip1) and the kinase ataxia-telangiectasia mutated (ATM) activities, two enzymes with essential roles in DNA repair and cell survival. Together, these findings are the first to identify the activation of signaling processes by IL-1β, leading to the acquisition of resistance to cisplatin in cancer cells.

## 2. Results

### 2.1. Interleukin-1β Induces Upregulation of Chemoresistance-Related Genes in 6D Cells

**Figure 1:**
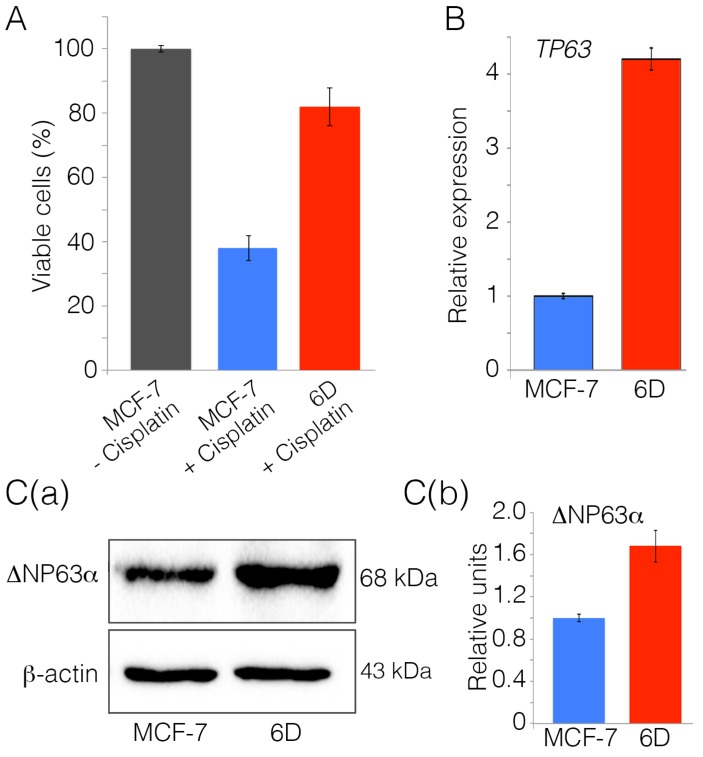
Interleukin 1β (IL-1β) induces resistance to cisplatin and upregulates the expression of *TP63*. (**A**) Non-invasive MCF-7 cells and invasive 6D cells were treated with 100 µM cisplatin (MCF-7 + cisplatin) and (6D + cisplatin). MCF-7 cells without any treatment were utilized as the control of live cells in the assay (100% viability). Cells were harvested at 48 h and quantification of viable cells performed using the WST-1 reagent. Data are presented as percentage ± SD of viable cells relative to untreated cells from three independent experiments. (**B**) Relative expression of the *TP63* gene was determined by qPCR in MCF-7 and 6D cells. Results represent the average of three independent experiments ± SD. (**C**) (a,b) Representative Western blot and densitometry analysis of total extracts from MCF-7 and 6D cells. The membranes were challenged with anti-TP63 antibody and anti-β-actin for protein load control. The densitometric analysis shows data in three blots from independent experiments. In all of them, ΔNp63α levels were normalized relative to the protein levels in MCF-7 cells. Asterisks indicate significance at *p* = 0.001.

Our previous results showed that cloned 6D cells acquired resistance to doxorubicin and tamoxifen, together with other features of aggressive cancer cells, through an EMT program induced by activation of the IL-1β/IL-1RI/β-catenin pathway [4,5,6]. To determine if these cells also acquire resistance to cisplatin, their viability was evaluated after exposure to the drug for 48 h. Figure 1A shows that, in parental MCF-7 cells exposed to cisplatin, the viability was reduced to less than 40%, while, in 6D cells in the same conditions, viability was 83%. Control viability, defined as 100%, was assigned to parental MCF-7 cells not exposed to cisplatin.

An RNA-seq analysis previously performed with 6D cells showed increased expression of *TP63*, a gene that, together with other human genes, is related to cell survival and chemoresistance [5]. We asked if the IL-1β-induced overexpression of TP63 in the 6D cells could be related to their resistance to cisplatin. Quantitative PCR analysis showed that, after IL-1β-stimulation, the expression of TP63 transcript in the 6D cells had a 4.2-fold increase relative to the baseline expression found in the parental MCF-7 cells (Figure 1B). To confirm whether IL-1β stimulation also induced upregulation of the TP63 isoforms, cell extracts from MCF-7 and 6D cells were compared by Western blot, using an antibody specific for all of the TP63 isoforms. The results in Figure 1C(a) revealed that, in parental MCF-7 and 6D cells, only one TP63 isoform is expressed in 6D cells. According to the electrophoretic pattern, this protein could correspond to the reported 68–72-kDa ΔNp63α isoform [15,16]. To confirm the identity of the expressed isoform, bioinformatic analyses were performed using RNA-seq data. The results shown in Figure S1 (Supplementary Materials) indicate that the ΔNp63α isoform was the most prominent isoform expressed in the 6D cells. Densitometric analysis of blots from three independent experiments showed that IL-1β stimulation increased 1.7-fold the levels of the ΔNp63α expressed in 6D cells compared to the levels found in the parental MCF-7 cells (Figure 1C(b)).

### 2.2. Silencing of ΔNp63α Decreases Resistance to Cisplatin in 6D Cells

**Figure 2:**
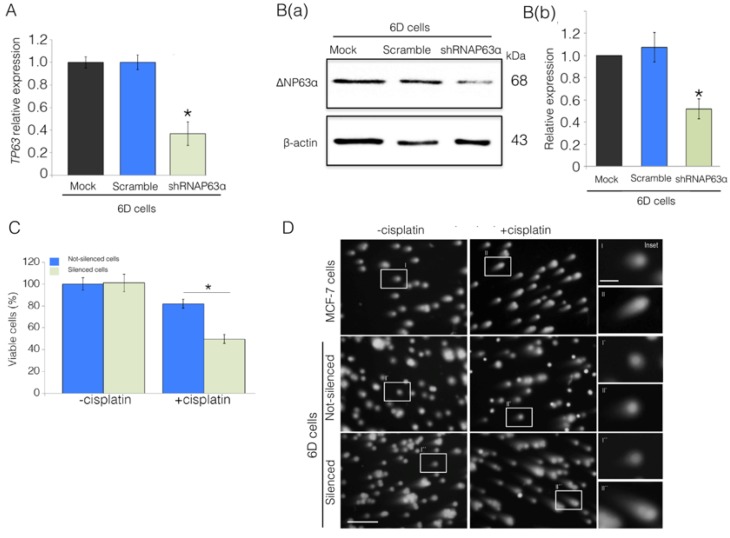
ΔNp63α plays a role in the IL-1β induction of cisplatin resistance in 6D cells. Cells were transfected with empty vector (Mock), non-specific short hairpin RNA (Scramble), and the specific silencing RNA (shRNAp63α). (**A**) *TP63* expression was evaluated by qPCR. Results represent the average of three independent experiments ± SD. Asterisks correspond to *p* = 0.001 relative to the controls, Mock and Scramble. (**B**(a)) Representative Western blot of ΔNp63α protein levels in the 6D cells. (**B**(b)) Densitometric values corresponding to ΔNp63α levels in (**B**(a)) were normalized to those of β-actin. (**C**) Cell viability levels determined in ΔNp63α-silenced and non-silenced cells in the absence or presence of cisplatin. Data represent the average of four independent experiments ± SD. Asterisks indicate significance relative to 6D cells at *p* = 0.001. (**D**) Comet assay to evaluate DNA integrity and damage by cisplatin in MCF-7, non-silenced, and shRNAp63α-silenced 6D cells. Cells not treated and treated with cisplatin were mixed with low-melting point agarose, lysed, and subjected to electrophoresis, followed by staining with ethidium bromide. DNA was visualized by fluorescence microscopy; scale bar = 100 μm. The insets in the right panel show magnified images taken from each condition; scale bar = 25 μm.

To determine the direct participation of ΔNp63α overexpression in the acquired resistance to cisplatin, a stable ΔNp63α-silenced cell line was obtained by transfection of 6D cells with the silencer short hairpin RNAp63α (shRNAp63α). The results in Figure 2A showed that expression of TP63 mRNA in the silenced cells decreased to 37%, relative to its expression in cells only transfected with the empty vector (Mock) or transfected with a non-specific shRNA (Scramble) in which the expression of *TP63* mRNA was not affected. Figure 2B(a) shows a representative blot of the extracts from non-silenced and silenced cells in which a reduction to 50% ΔNp63α levels were detected in the ΔNP63α silenced cells. The densitometric analysis from three independent experiments is shown in Figure 2B(b).

The relationship between downregulation of ΔNp63α and decreased resistance to cisplatin became clear when viability of ΔNp63α silenced cells exposed to the drug decreased to 50%, while, in the same conditions, non-silenced cells maintained a viability of 83% (Figure 2C). A comet assay, designed to reveal DNA damage by formation of comet tails at the level of individual cells (Figure 2D) showed that cisplatin caused significant DNA harm in MCF-7 and the ΔNP63α-silenced 6D cells, but not in the drug-resistant 6D cells. In addition, immunofluorescence assays, using an antibody to the phosphorylated histone γH2AX to visualize the early damage of DNA caused by cisplatin, showed high expression of γH2AX in parental MCF-7 and in the silenced-6D cells exposed to the drug but not in non-silenced 6D cells (Supplementary Figure S2, Supplementary Materials).

These results suggest that the IL-1β stimulus, through upregulation of TP63 and ΔNp63α, is an inducer of the resistance to cisplatin.

### 2.3. ΔNp63α Modulates the Expression and Activation of Survival Gene EGFR

**Figure 3:**
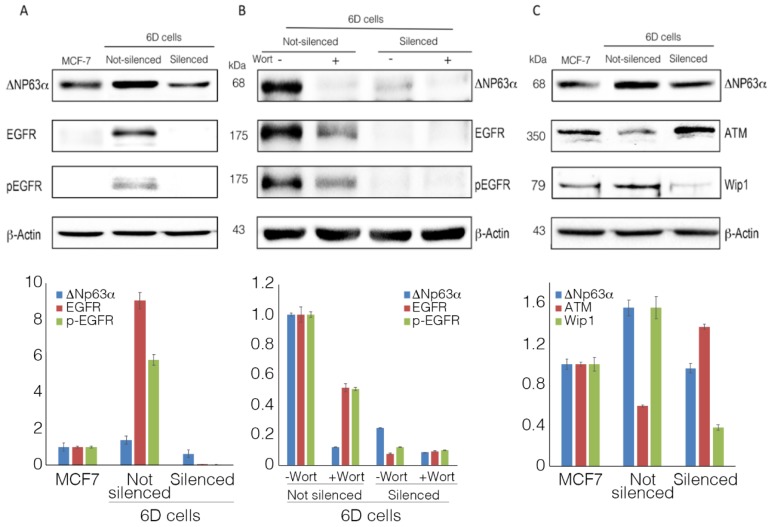
ΔNp63α is essential for the expression of epidermal growth factor receptor (EGFR) and phosphatase 1D (Wip1). (**A**) Representative Western blot of EGFR expression and its activated form pEGFR (phosphorylated at Tyr1068) in parental MCF-7cells and in non-silenced and shRNAp63α-silenced 6D cells. (**B**) Wortmannin’s inhibitory effect on ΔNp63α and EGFR overexpression and phosphorylation of the latter. (**C**) Western blot analysis of ataxia-telangiectasia mutated (ATM) and Wip1 expression in the cells indicated in panel A. Densitometric analyses of Western blots show the results of at least three independent experiments.

It was suggested that the isoform ΔNp63α could regulate the expression of the epidermal growth factor receptor (EGFR), a molecule involved in the expression of malignancy markers, proliferation, cell survival, and chemoresistance [17,18]. The phosphorylation of this receptor in different cancers is known to activate phosphoinositide 3-kinase (PI3K)/protein kinase B (AKT)-dependent pathways that participate in diverse signaling that promotes proliferation and resistance to chemotherapy [19]. As previously reported in our studies with 6D cells, PI3K and AKT are important effectors in the IL-1β/IL-1RI/β-catenin signaling pathway, which leads to acquisition of resistance to doxorubicin and tamoxifen [4,5,6]. Therefore, we investigated if ΔNp63α upregulation, induced by IL-1β, could trigger the expression and activation of EGFR and if this had an effect on the cell resistance to cisplatin. The Western blot in Figure 3A shows the basal expression of ΔNp63α, the very low levels of EGFR, and the almost null presence of its phosphorylated form (pEGFR) in the parental MCF-7 cells, while, in non-silenced cells, ΔNp63α, EGFR, and pEGFR levels were significantly increased. In contrast, the levels of the proteins in ΔNp63α silenced cells decreased, and EGFR and pEGFR were not detected. Moreover, when cells were treated with wortmannin, a specific inhibitor of PI3K/AKT activity in the IL-1β pathway [4], the increased expression of ΔNp63α, EGFR, and pEGFR observed in non-silenced cells was greatly reduced after treatment with the inhibitor (Figure 3B). In the silenced cells, even the residual levels of ΔNp63α and EGFR were also decreased by wortmannin.

These results show that the expression of ΔNp63α caused an upregulation and phosphorylation of EFGR via the activation of the IL-1β/IL-1RI/β-catenin signaling pathway. The data also support the hypothesis that these pathways are linked to the signaling processes leading to resistance to cisplatin.

### 2.4. ΔNp63α Modulates the Levels of Proteins ATM and Wip1

The expression of ΔNp63α is associated with ATM and its function in sensing DNA damage and apoptosis. The possible involvement of ATM regulation in the cell resistance to cisplatin was analyzed. Figure 3C shows that, in wild-type 6D cells (resistant to cisplatin), overexpression of ΔNp63α concurred with a considerable decrease in the expression of ATM. In contrast, in ΔNp63α-silenced cells (sensitive to cisplatin), ATM levels were greatly increased. These results indicate that overexpression of ΔNp63α directly or indirectly inhibits the expression of ATM. To evaluate the participation of Wip1, the levels of this phosphatase, a known regulator of the kinase ATM [20], were evaluated as a possible intermediary between ATM and ΔNp63α. Figure 3C shows that, when ΔNp63α was overexpressed, Wip1 levels increased, causing the drop of ATM, whereas when ΔNp63α was silenced and cells became sensitive to cisplatin, Wip1 levels decreased and ATM levels augmented. These results show that ΔNp63α overexpression participates in the regulation of these proteins.

## 3. Discussion

The present study shows, for the first time, that the inflammatory IL-1β is an inducer of chemoresistance to cisplatin through activation of the IL-1β/IL-1RI/β-catenin signaling pathway that we described previously as operating in the induction of expression of genes associated with cell survival and resistance to anti-cancer drugs [5,6]. Triggering of this pathway induced the expression of high levels of ΔNp63α via PI3K/AKT activation. The overexpression of ΔNp63α could activate the receptor EGFR into its phosphorylated form via a positive feedback stimulation, promoting the PI3K/AKT activity and the downstream continuity of the IL-1β signaling. The link between high levels of ΔNp63α and the activation of EGFR was demonstrated using wortmannin, a specific inhibitor of PI3K/AKT activities, whereby blocking the operation of the IL-1β pathway caused a significant drop of ΔNp63α and EGFR levels. These processes were validated when non-silenced 6D cells were treated with wortmannin, as ΔNp63α and EGFR levels were diminished in this condition.

It was also observed that ΔNp63α upregulation occurred together with the increase of protein Wip1 levels, which in turn downregulated the expression of ATM, allowing DNA repair, survival of the cells, and resistance to cisplatin. In contrast, when ΔNp63α was silenced, Wip1 levels were drastically reduced and ATM levels were increased; similar results were reported knocking down Wip1 [21]. In both cases, when ΔNp63α was silenced and Wip1 was inhibited, the cells became sensitive to cisplatin.

Cisplatin is a selective drug in the treatment of metastatic breast cancer [8]. Nevertheless, a significant proportion of patients eventually develop resistance to this drug, leading to tumor relapse and limiting its clinical usefulness [22]. Reports in the literature provided information for the possible role of the *TP63*-encoded protein ΔNp63α in the acquisition of resistance to cisplatin [7,23]. Our previous RNA-seq analysis of IL-1β-stimulated 6D breast cancer cells showed increased expression of *TP63*, a gene classified in the group of drug-resistance-related genes [5].

β-catenin translocation and its binding to transcription factor/lymphoid enhancer-binding factor (TCF/LEF) sites (present in the P2 promoter of *TP63*) were described as key regulators of ΔNp63α expression [14]. Our previous studies with 6D cells demonstrated that activation of the IL-1β/IL-1RI/β-catenin signaling pathway by the inflammatory cytokine IL-1β elicits nuclear translocation of β-catenin. Once in the nucleus, this protein increased the transcriptional activity of several genes associated with malignancy markers, including *BIRC3* and *ESR1.* The changes in the expression of these genes were involved in the acquired resistance to anti-cancer drugs, doxorubicin and tamoxifen, respectively [4,5,6]. Therefore, we analyzed the pathway induced by IL-1β and found that this pathway was involved in ΔNp63α upregulation and the cell resistance to cisplatin.

It was reported that, in head and neck cancer cells transfected to overexpress ΔNp63α, upregulation of genes associated with apoptosis and DNA repair occurs, as well as increased activity of survival proteins [7,23,24]. The participation of AKT signaling pathways was suggested as an intermediary in the activities reported associated with the resistance to cisplatin [25].

**Figure 4:**
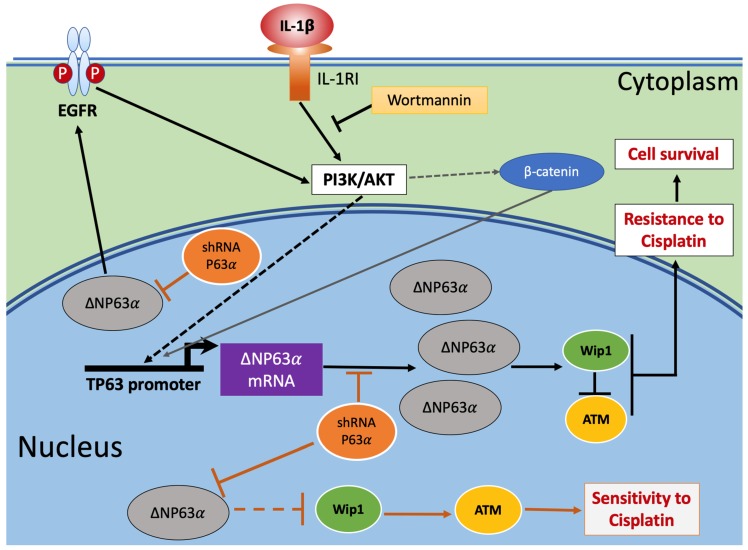
Graphical model of IL-1β activation of signaling pathways leading to acquisition of resistance to cisplatin in breast cancer cells. Triggering of the IL-1β/IL-1RI/β-catenin pathway by IL-1β induces the activity of downstream effectors phosphoinositide 3-kinase (PI3K)/protein kinase B (AKT) [4], leading to translocation of β-catenin to the nucleus and regulation of proteins that participate in the acquisition of resistance to cisplatin. The induced upregulation of ΔNp63α regulates the expression of cell survival (pEFGR) and DNA damage response (Wip1 and ATM) proteins. At the same time, the increased levels of ΔNP63α activate EGFR, which, through a feed-back loop, maintains PI3K/AKT activity and the continuity of the downstream signaling initiated by IL-1β, which leads to resistance to cisplatin. However, if ΔNP63α overexpression is hampered by silencing its mRNA (shRNAP63α), the lower levels of ΔNP63α expressed in the cells will not be sufficient to maintain high levels of Wip1, thereby increasing ATM levels and the sensitivity to cisplatin. Dashed arrows indicate indirect positive modulation in the pathway that was previously reported [4,14]. The link between low levels of ΔNp63α and Wip1 is represented in the figure with a dashed line because the step(s) leading to decreased levels of Wip1 are not yet well identified.

Figure 4 integrates our previously published data [4] and the present results into a graphical model that highlights tested and proposed mechanisms which could explain the induction of resistance to cisplatin in 6D breast cancer cells.

The findings are the first to show that IL-1β is an inducer of signaling pathways in which overexpression of ΔNp63α leads to resistance to cisplatin.

## 4. Materials and Methods

### 4.1. Cell Culture

The MCF-7 cells (ATCC, Manasas, VA, USA) and the 6D cells, a clone selected from MCF-7 cells highly responsive to IL-1β stimulus, were cultured in Dulbecco’s modified Eagle medium (DMEM-F12) supplemented with 10% fetal bovine serum (FBS), penicillin (5000 U/mL), and streptomycin (5000 μg/mL) from Gibco BRL (Grand Island, NY, USA). Cultures were incubated at 37 °C with 5% CO_2_. For all the experiments, the 6D cells were re-stimulated with 20 ng/mL human recombinant IL-1β (Peprotech, Rocky Hill, NJ, USA) for 48 h to ensure their homogeneous response to IL-1β [4].

### 4.2. Gene Expression

Total RNA was extracted from the cells using Trizol^®^ (Invitrogen, Carlsbad, CA, USA). One microgram of RNA was used for reverse transcriptase reactions. Gene expression was carried out using the Fast Start SYBR Green Master kit (Applied Biosystems, Foster City, CA, USA), using a 7500 Real-Time Thermal Cycler (Applied Biosystems). Relative gene expression values were normalized to the constitutive expression of the *RPLP0* gene encoding 60S acidic ribosomal protein P0. Values were determined using the 2^−ΔΔCT^ method [26]. The specific primers for RPLP0 [4] and TP63 [27] are shown in Table S1 (Supplementary Materials). 

### 4.3. SDS-PAGE and Western Blotting

Protein extracts were obtained from cell lysates with radioimmunoprecipitation assay (RIPA) 1× buffer supplemented with Complete^®^ inhibitors cocktail (Roche Applied Science, Mannheim, Germany). Proteins were separated by 10% SDS-PAGE and blotted onto nitrocellulose membranes and blocked. The membranes were then exposed to mouse anti-human TP63 (1:1500) or WIP1 (1:1000) antibodies (both from GeneTex, Irvine, CA, USA), anti-EGFR (1:1000), anti-p-Try1068-EGFR (1:1000), or anti-ATM (1:1000) antibodies (all from Cell Signaling Technology, Danvers, MA, USA). The anti-β-actin monoclonal antibody, kindly donated by Dr. JM Hernández (CINVESTAV-IPN), was utilized to detect cell actin as the protein load control. Horseradish peroxidase (HRP)-tagged secondary antibodies anti-rabbit or anti-mouse (1:5000) (Jackson Immunoresearch, West Grove, PA, USA) were used for chemiluminescent detection with the Immobilon™ Western Chemiluminescent HRP Substrate kit (Millipore, MA, USA). Chemiluminescence signals were recorded on a ChemiDoc imaging device (Bio-Rad Laboratories, Hercules, CA, USA) and densitometric analyses were performed with ImageJ software.

### 4.4. Resistance to Cisplatin

Fifty thousand cells per well were seeded in 96-well culture plates and incubated for 24 h, then switched to medium with only 1% FBS for 18 h. After this, cells were treated with different conditions: (1) incubated with 100 μM cisplatin (PISA Pharmaceutics, Guadalajara, Mex.); (2) incubated in DMEM-F12 containing 100 μM cisplatin and 20 ng/mL IL-1β; (3) incubated only in culture medium as a control. All the groups were incubated for 48 h at 37 °C. To quantify cell viability for each condition at the indicated time, the WST-1 assay (Roche Applied Science, Mannheim, Germany) was used as described previously [5].

### 4.5. Silencing of 6D Cells With shRNAp63α

A stable 6D knockdown cell line was prepared using a silencer reported and validated to silence all the isoforms of TP63 [28]. For the production of lentivirus containing the shRNA shp63α pLKO.1 with a puromycin-resistance cassette, Addgene plasmid 19120 [28] or the scrambled non-specific hairpin shRNA control, pLKO.1 shSCR, Addgene plasmid 17920 [29], the manufacturer’s instructions were followed. Briefly, HEK 293T cells were transfected at a confluence of 70% using serum-free DMEM containing 9 μg of each packaging plasmid (psPAX2 and pMD2.G; Addgene plasmids 12260 and 12259, respectively) and 15 μg/mL polyethyleneimine. Virus-containing media were collected 24 and 48 h post-transfection. The 6D cells were transduced with a multiplicity of infection (MOI) of 10 for 4 h at 37 °C and then incubated for 24 h with fresh medium. To select for infected cells, the medium was replaced with fresh medium containing 5.0 μg/mL puromycin. The silencing of the TP63 isoform present in 6D cells was confirmed by qPCR and Western blotting before further experiments. The viability of the transfected cells was evaluated after cisplatin was added to the cultures for 48 h, as indicated in the WST-1 assay.

### 4.6. DNA Damage and Repair

For individual cell characterization of DNA damage by cisplatin, the comet assay was carried out following the neutral lysis method, as previously described [30]. The DNA comet tails formed in cisplatin-damaged cells were analyzed by fluorescence microscopy. DNA damage by cisplatin was also evaluated in MCF-7, 6D, and ΔNP63α-silenced cells by immunofluorescence as previously reported [4]. A specific antibody (NovusBIO, Littleton, CO, USA) directed to the phosphorylated histone ϒH2XA (pSer139) and a secondary antibody anti-rabbit immunoglobulin G (IgG), tagged with Alexa 488, were utilized to assess the histone association with damaged DNA. Nuclei were stained with 4′,6-diamidino-2-phenylindole (DAPI). All the images were acquired with a digital camera Olympus DP72 coupled to an epifluorescence microscope (Olympus 50× model).

### 4.7. Statistical Analysis

Data are presented as means ± SD. The R software for statistical computing (v 3.3.3) with the Base package was used for statistical analysis. For comparisons between two data groups, the Mann–Whitney U test was applied; whereas, for comparisons between three or four data groups, the Kruskal–Wallis test was applied. Significant differences were established when the *p*-value was equal to or lower than 0.05.

## Supplementary Materials

Supplementary Materials [31,32,33] can be found at http://www.mdpi.com/1422-0067/20/2/270/s1.

## Abbreviations

TP63	Tumor protein p63
ATM	Ataxia-telangiectasia mutated protein
Wip1	Protein phosphatase 1D
EGFR	Epidermal growth factor receptor
EMT	Epithelial–mesenchymal transition
MOI	Multiplicity of infection
