# Supplementary material for: IL-1β Inflammatory Cytokine-Induced TP63 Isoform ∆NP63α Signaling Cascade Contributes to Cisplatin Resistance in Human Breast Cancer Cells
Source: Int J Mol Sci. 2019 Jan 11;20(2):270. doi: 10.3390/ijms20020270 (PMC6358904; doi:10.3390/ijms20020270)
Supplement: Supplementary file 1 [file ijms-20-00270-s001.pdf]

Supplementary Material

IL-1β inflammatory cytokine induced TP63 isoform ΔNP63α signaling cascade, contributes to cisplatin resistance in human breast cancer cells

Mónica G. Mendoza-Rodríguez, Jorge T. Ayala-Sumuano, Lázaro García-Morales, Horacio Zamudio-Meza, Eloy A. Pérez-Yepez and Isaura Meza

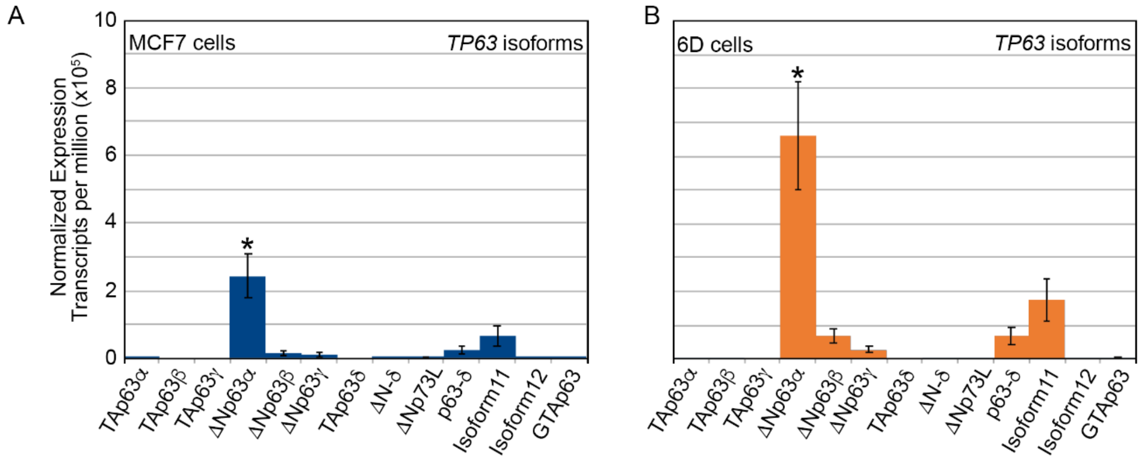

Supplementary Figure S1. Differential expression analysis of *TP63* isoforms. RNA-seq data from MCF7 (A) and 6D (B) cells were aligned to human genome version GRCh38 using TopHat2 (v 2.1.1) [31,32]. For differential expression of the 13 TP63 isoforms in MCF7 and 6D cells, all reads from RNA-seq data that aligned to the genomic region corresponding to the coordinates of *TP63* gene were realigned against a reference that contains the sequence of every of the transcript variants known for this gene with TopHat2. The expression levels for each isoform was determined by Salmon (v 0.12.0) [33]. Data was normalized as transcripts per million, relative to the whole number of aligned reads for each condition from the RNA-seq dataset. Statistical differences were established by Fisher's Exact Test and asterisks indicate statistical differences in the expression of ΔNp63α in 6D cells relative to the same isoform in MCF7 cells with a P value equal to 0.003.

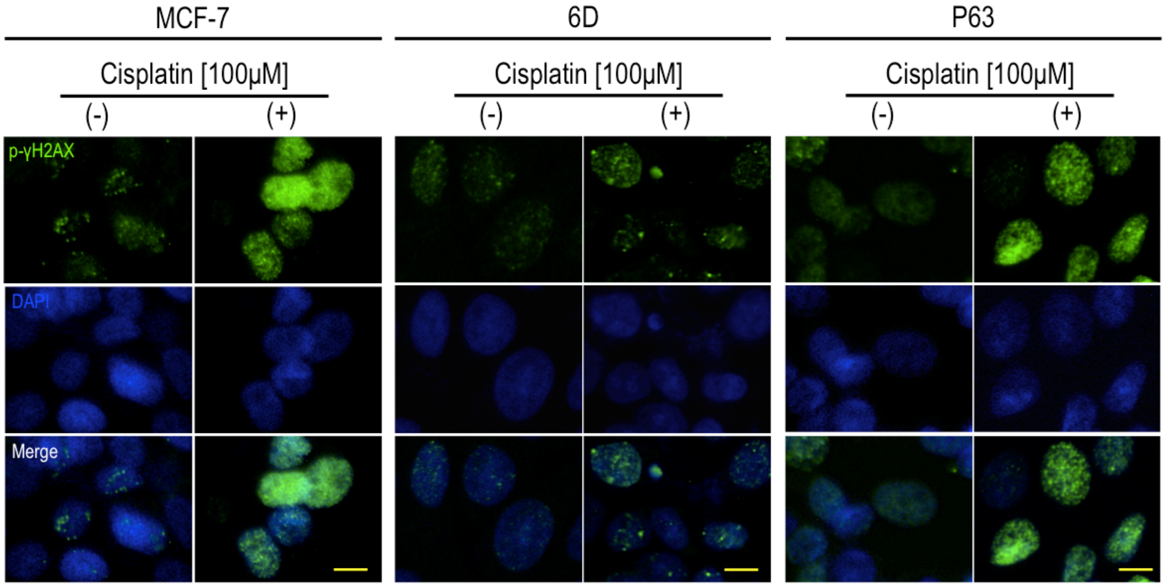

Supplementary Figure S2. Nuclear distribution of phosphorylated histone γH2AX after cisplatin treatment of breast cancer cells. Parental MCF-7 cells, and 6D not-silenced and silenced cells (P63) were treated with 100 μM of cisplatin for 24 h. Cells were stained with the antibody to the phosphorylated γH2AX histone (p Ser139) and a secondary antibody tagged with Alexa 488 (green). Nuclei were stained with DAPI (Blue). Representative images from 10 randomly chosen fields of each experimental condition showed pγH2AX confined to the nuclei, forming few clusters or foci in cells not-treated with cisplatin. Significant increase of the histone labeling was observed in MCF-7 and silenced P63 cells treated with cisplatin. In contrast, 6D cells did not show increased labeling of the pH2AX after treatment with the drug. Bar = 20μm.

Supplementary Table S1. Primers used for relative quantitative RT-PCR analysis.

Gene	Primer Bank ID	Primer Sequence	Ta (°C)	Source
RPLP0	None	5’- AGC CCA GAA CAC TGG TCT C-3’<br>5’- ACT CAG GAT TTC AAT GGT GCC-3’	60	[4]
TP63	169234656C3	5’-GTC ATT TGA TTC GAG TAG AGG GG-3’<br>5’-CTG GGG TGG CTC ATA AGG T-3’	60	[27]
